# Obesity and Cytokines in Childhood-Onset Systemic Lupus Erythematosus

**DOI:** 10.1155/2014/162047

**Published:** 2014-03-03

**Authors:** Nailú Angélica Sinicato, Mariana Postal, Fernando Augusto Peres, Karina de Oliveira Peliçari, Roberto Marini, Allan de Oliveira dos Santos, Celso Dario Ramos, Simone Appenzeller

**Affiliations:** ^1^Faculty of Medical Science, State University of Campinas, Cidade Universitaria, 13083-970 Campinas, SP, Brazil; ^2^Rheumatology Unit, Department of Medicine, Faculty of Medical Science, State University of Campinas, Cidade Universitária, 13083-970 Campinas, SP, Brazil; ^3^Department of Pediatrics, Faculty of Medical Science, State University of Campinas, Cidade Universitária, 13083-970 Campinas, SP, Brazil; ^4^Department of Radiology, Nuclear Medicine Division, State University of Campinas, Cidade Universitaria, 13083-970 Campinas, SP, Brazil

## Abstract

*Background*. In systemic lupus erythematosus (SLE), atherosclerosis is attributed to traditional and lupus related risk factors, including metabolic syndrome (MetS), obesity, and inflammation. *Objective*. To evaluate the association between obesity, measures of body fat content, serum tumor necrosis factor alpha (TNF-*α*), and interleukin (IL)-6 and -10 levels in childhood-onset SLE (cSLE). *Methods*. We screened consecutive cSLE patients followed up in the Pediatric Rheumatology Outpatient Clinic of the State University of Campinas. cSLE patients were assessed for disease and damage. Obesity was definite as body mass index (BMI) ≥30 kg/m^2^. Serum TNF-*α*, IL-6, and IL-10 levels were measured by ELISA. Dual-energy X-ray absorptiometry was used to determine total fat mass, lean mass, and percent of body fat. *Results*. We included 52 cSLE patients and 52 controls. cSLE patients had higher serum TNF-*α*  (*P* = 0.004), IL-6 (*P* = 0.002), and IL-10 (*P* < 0.001) levels compared to controls. We observed higher serum TNF-*α*  (*P* = 0.036) levels in cSLE patients with obesity. An association between serum TNF-*α* levels and body fat percent (*P* = 0.046) and total fat mass on trunk region (*P* = 0.035) was observed. *Conclusion*. Serum TNF-*α* levels were associated with obesity and body fat content in cSLE. Our finding suggests that obesity may contribute to the increase of serum TNF-*α* levels in cSLE.

## 1. Introduction

Systemic lupus erythematosus (SLE) is a chronic systemic inflammatory disease affecting mainly women during childbearing age [1]. Although life expectancy has improved significantly, no changes in morbidity and mortality related to cardiovascular disease (CVD) have been observed in SLE patients in the past decades [2, 3]. In addition to traditional risk factors, many lupus-specific factors are linked to the increased risk of CVD observed in SLE [4–6].

Obesity-associated systemic inflammation is characterized by increased circulating proinflammatory cytokines and activation of several kinases that regulate inflammation [7–9]. Recent evidence supports that obesity-induced inflammation is mediated primarily by immune cells such as the macrophages and T lymphocytes present in metabolic tissues [9]. Adipose tissue derived cells can produce inflammatory cytokines, such as tumor necrosis factor alpha (TNF-*α*), interleukin (IL) 6, and IL-10 [10, 11].

TNF-*α* and IL-6 are proinflammatory cytokines associated with an increased insulin resistance, inhibition of insulin receptor autophosphorylation, and signal transduction. These mechanisms lead to insulin resistance, hyperglycemia, and dyslipidemia [12–18]. IL-10 is also known as an antiatherogenic cytokine. Upregulation of IL-10 locally or systemically reduces atherosclerosis development in mouse models [13–15].

The aim of this study was to evaluate the association between obesity, measures of body fat content, and serum TNF-*α*, IL-6, and IL-10 in cSLE.

## 2. Patients and Methods

### 2.1. Subjects

Fifty-two consecutive cSLE patients, recruited from the Pediatric Rheumatology Outpatient Clinic of the State University of Campinas were included in this study. Patients were included in the present study if they (i) fulfilled at least four criteria of the American College of Rheumatology (ACR) [19]; (ii) were below 18 years of age at disease onset; and (iii) had a follow-up duration of at least 6 months (time necessary to evaluate damage index).

Fifty-two healthy volunteers (caregivers or students) matched by age, gender, and sociodemographic characteristics were included as a control group. None of the controls had any history of chronic disease, including autoimmune diseases.

This study was approved by the ethics committee at our institution, and the informed written consent was obtained from each participant and/or legal guardian.

### 2.2. Clinical Features

All patients had their medical histories and clinical,and serological characteristics entered at the time of cSLE diagnosis into special computer database programs. Features included in this protocol were age at the onset of disease (defined as the age at which the first symptoms clearly attributable to SLE occurred), age at diagnosis (defined as the age when patients fulfilled four or more of the 1987 revised criteria for the classification of SLE [19]), and follow-up time (defined as the time from disease onset until December 2012).

Total doses and length of use the of corticosteroids since the onset of disease were calculated by careful review of the medical charts. Doses of oral and parenteral corticosteroids were converted to the equivalent doses of prednisone. The cumulative dose of corticosteroids used was calculated by the sum of the daily dosages versus the time (days) of treatment. We also calculated the cumulative corticosteroid dose adjusted by weight by summing up the daily corticosteroid dose per weight at each routine visit.

### 2.3. Disease Activity and Cumulative Damage

Disease activity was measured by the Systemic Lupus Erythematosus Disease Activity Index (SLEDAI) [20]. SLEDAI scores range between 0 and 105, and the scores of ≥3 were considered as active disease [21]. Adjusted SLEDAI scores over time were calculated by careful review of the medical charts and preview exams [22]. Cumulative SLE-related damage in all patients was determined by using the Systemic Lupus International Collaborating Clinics (SLICC)/ACR Damage Index (SDI) [23].

### 2.4. Body Mass Index

Body mass index (BMI) was calculated as weight (kg) divided by height (m) squared (kg/m^2^). Criteria used to define nutritional status were based on the World Health Organization (WHO) criteria [24]. BMI cutoff points for Brazilian children and adolescents were used for individuals between 2 and 18 years [25]. Obesity was considered when BMI was above 30 Kg/m^2^.

### 2.5. Dual X-Ray Absorptiometry (DXA)

Percentual body fat (PBF), fat mass, and lean mass were obtained by DXA scan (Hologic Discovery Wii), through Whole Body Auto Fan Beam. This scan determines total fat mass and total lean mass in kilograms in addition to total fat mass and total lean mass as a percentage of total body mass.

### 2.6. Blood Sampling

Blood samples were collected from peripheral veins of all individuals in dry tubes and left to clot at room temperature for 30 minutes. Blood samples were then centrifuged for 15 minutes at 3000 rpm, and the serum was then stored in aliquots at −80°C for future use. We did not collect blood samples from individuals during an episode of acute or chronic infection.

### 2.7. Cytokine Assay

Commercially available kits from R&D Systems (London, UK) were used for the measurement of serum TNF-*α*, IL-6, and IL-10 levels by enzyme-linked immunosorbent assay (ELISA), carried out in accordance with the manufacturer's instructions. The minimum detectable dose (MDD) was 0.106 pg/mL for TNF-*α*, 0.039 pg/mL for IL-6, and 3.9 pg/mL for IL-10.

### 2.8. Statistical Analysis

All the data were tested for their normal distribution (Kolmogorov-Smirnov test). Categorical variables were compared by *χ* ^2^ test. Nonnormal variables were compared by Fisher exact tests. Mann-Whitney *U* test was used to compare anthropometric measure and laboratory studies between patients and controls. Spearman's correlation was used to correlate continuous variables (e.g., TNF-*α* levels, SLEDAI, and SDI scores). For all analyses, *P* value ≤ 0.05 was considered to be statistically significant. Statistical analysis was carried out using IBM SPSS Statistics 16.0 software (SPSS/IBM, Chicago, IL, USA).

## 3. Results

### 3.1. Demographics

We included 52 consecutive cSLE patients. Forty-seven (90.3%) were women with mean age of 17.6 years (standard deviation (SD) ± 3.7 years). Mean disease duration was 5.14 years (SD ± 4.05). The control group consisted of 52 controls (47 women) with mean age of 18.2 years (SD ± 6.4). Patients and healthy controls were statistically comparable in terms of age and sex (Table 1).

### 3.2. BMI Analyses

BMI was similar between patients (median 21.74 kg/m^2^; range: 16.1–31.12 kg/m^2^) and controls (median 21.43 kg/m^2^; range: 14.36–28.54 kg/m^2^) (*P* = 0.101). Sixteen (31%) cSLE patients were overweight compared to 6 (11.5%) controls (*P* = 0.018).

We did not observe an association between BMI and SLEDAI, SDI, and cumulative corticosteroid dose.

### 3.3. Body Composition Analysis

On whole body analysis, we observed a median fat mass of 22.38 kg (range: 7.67 kg–36.62 kg), a median lean mass of 35.49 kg (range: 25.31 kg–52.14 kg), and a median PBF of 34.1% (range: 12.1–54.4%) in cSLE. In the trunk region we observed a median fat mass of 8.62 kg (range 2.98 kg–17.59 kg), median lean mass of 16.80 kg (range: 11.24 kg–26.19 kg) and a PBF of 42.3% (range: 12.1–54.4%).

### 3.4. Cytokine Assay

Serum TNF-*α* (*P* = 0.004), IL-6 (*P* = 0.002), and IL-10 (*P* < 0.001) levels were significantly increased in cSLE patients when compared to healthy controls (Table 2). We observed higher serum TNF-*α* levels in obese cSLE patients when compared with nonobese cSLE patients (*P* = 0.036), obese controls (*P* = 0.039) and non-obese controls (*P* < 0.0001) (Table 3). No difference in serum TNF-*α* levels was observed between obese and non-obese healthy controls (*P* > 0.05). We observed an association between TNF-*α* and PBF (*P* = 0.046) and total fat mass on trunk region (*P* = 0.035) analyzed by DXA scans.

No association between serum IL-6 and IL-10 levels and SLEDAI or SDI scores was observed. In addition, no difference in these cytokine levels in cSLE patients and controls with and without obesity was observed.

## 4. Discussion

Adipose tissue is known to be capable of secreting cytokines such as TNF-*α*, IL-6, and IL-10. Therefore, the purpose of this study was to assess whether the levels of these cytokines were increased in obese cSLE when compared to nonobese cSLE and healthy controls.

The observation that obese cSLE patients had higher serum TNF-*α* levels when compared to nonobese cSLE and healthy controls is the major finding of our study. In addition, we observed that serum TNF-*α* levels correlated with PBF and total fat mass in trunk region in cSLE.

Recent studies have demonstrated that increased adipose tissue mass contributes towards an increase in chronic inflammation [26, 27]. Chronic inflammation is further enhanced by inflammatory markers produced in the liver and in other organs [28]. Recently, it has been demonstrated that obesity is associated with a low-grade inflammatory process, characterized by increased circulating levels of proinflammatory cytokines such as TNF-*α*, IL-6, and acute-phase proteins (CRP) [29–32]. The mechanism underlying increased inflammation in the setting of obesity remains unclear, but it is known that mononuclear cells are activated and proinflammatory cytokines are upregulated in obese individuals [33, 34].

We observed an association between serum TNF-*α* levels and PBF and total fat mass in trunk region. Studies analyzing the association between serum TNF-*α* and DXA scans have not been reported in cSLE so far, but studies on healthy women and type-2 diabetes patients showed an association between plasma levels of TNF-*α* and visceral adipose tissue volume measured by CT-scan [35–38]. Previous studies have shown that visceral fat accumulation is associated with increased risk of CV risk [37]. In addition, with an increase in TNF-*α*, a reduction in lipoprotein lipase activity in adipose tissue is observed [39]. There is also evidence that TNF-*α* has a local effect, regulating adipocyte size in the face of increasing energy consumption [40, 41].

Cytokines, such as TNF-*α* and IL-6, are primarily involved in the early stages of the inflammatory response culminating in atherosclerosis [39, 42]. Increased TNF-*α* levels in the endothelium promote initial atheroma plaque [39, 42]. However, so far, studies were not able to conclude whether TNF-*α* is a causative factor of atherosclerosis.

Both IL-6 and TNF-*α* are expressed and secreted by human adipose tissue [43]. In obesity, increased secretion of IL-6 may contribute to metabolic dysfunction [44, 45]. In addition, one previous study has shown that IL-6 correlated positively with BMI and with measures of insulin resistance in abdominal obese male subjects [45]. As previously described in adults SLE patients, we observed higher IL-6 and IL-10 levels in cSLE patients when compared to healthy controls [46–49]. However, no association with BMI was observed in our cSLE cohort.

IL-10 downregulates inflammatory activation of monocytes and macrophages by transcriptional and posttranscriptional inhibition of the entire range of proinflammatory cytokines [50]. IL-10 has been shown to reduce atherosclerosis and it can be found in atheromatous plaque due to local macrophages production [50]. However, IL-10 is involved in SLE pathogenesis and it is increased in SLE patients with CVD compared to SLE patients without CVD [51, 52]. In our study, we did not observe an association between sera IL-10 levels and obesity.

We also did not observe an association between sera IL-6 levels and obesity. In the literature, it has been described that plasma IL-6 levels are associated with increased CV risk and observed in SLE patients with metabolic syndrome [53] and in patients with type 2 diabetes [44, 54]. In a large healthy family population study where children were included, IL-6 levels were closely associated with traditional and nontraditional risk factors for atherosclerosis [55].

Although cSLE is rare, it is important to consider that one limitation of our study is the small number of patients and controls included.

Corticosteroids are associated with weight gain due to increased appetite and fluid retention. Corticosteroids also cause a redistribution of fat deposition, occurring predominantly in the trunk and face [56–59]. However, we did not observe an association between serum TNF-*α*, IL-6, and IL-10 levels and corticosteroid dose.

To the best of our knowledge, this is the first study to evaluate the association of BMI, body composition and serum TNF-*α*, IL-6, and IL-10 levels in cSLE patients. Although these cytokines have been shown to be associated with CVD in other populations, we only observed an association between serum TNF-*α* levels and obesity, and PBF and total fat mass in trunk region. Our findings suggest that total fat mass may contribute to increased levels of serum TNF-*α* levels in cSLE.

## Figures and Tables

**Table 1:** Demographics data from cSLE and controls.

	cSLE patients *N* = 52	Healthy controls *N* = 52
Age (mean ± SD)	17.6 ± 3.7	18.2 ± 6.4
Female (*N*; %)	47 (90.3)	47 (90.3)
Disease duration (mean ± SD)	5.14	—

**Table 2:** Sera cytokines levels of the individuals included in the study.

Sera levels	cSLE patients N = 52	Healthy controls *N* = 52
TNF-*α*	1.93 pg/mL* (0.8–11.17 pg/mL)	1.23 pg/mL (0.25–3.91 pg/mL)

IL-6	1.46 pg/mL* (0.34–9.74 pg/mL)	0.95 pg/mL (0.39–3.91 pg/mL)

IL-10	13.86 pg/mL* (3.93–56.92 pg/mL)	6.64 pg/mL (3.52–9.54 pg/mL)

*P ≤ 0.05.

The data were given in median (range).

cSLE: childhood-onset systemic lupus erythemathosus; TNF-*α*: tumor necrosis factor alpha; IL: interleukin.

**Table 3:** Cytokines levels and therapy information from subjects subdivided into obese and nonobese.

	Obese cSLE	Nonobese cSLE	Obese controls	Nonobese controls
	N = 16	N = 36	*N* = 7	N = 45
Cytokines levels				
TNF-*α* (pg/mL)	3.1 (1–11.1)*	1.8 (0.8–11.1)	1.3 (0.5–2.1)	1.2 (0.2–3.9)
IL-6 (pg/mL)	1.4 (0.3–6.9)	1.4 (0.3–9.7)	0.9 (0.4–5.9)	0.9 (0.3–3.6)
IL-10 (pg/mL)	16.7 (7.6–26.3)	13.6 (3.9–39.7)	4.9 (3.9–6)	5.6 (3.5–9.5)
Therapy				
CE dose (mean ± SD)	17.3 ± 19.8	18.3 ± 19.8	—	—
CE/Kg (mean ± SD)	535.1 ± 339.5	444.5 ± 245.9		
CE cumulative (mean ± SD)	28036.7 ± 17611.5	23057 ± 16568.7		

The cytokine data were given in median (range). *P < 0.05.
